# Exome sequencing of UK birth cohorts

**DOI:** 10.12688/wellcomeopenres.22697.2

**Published:** 2024-12-05

**Authors:** Mahmoud Koko, Laurie Fabian, Iaroslav Popov, Ruth Y. Eberhardt, Gennadii Zakharov, Qin Qin Huang, Emma E. Wade, Rafaq Azad, Petr Danecek, Karen Ho, Amy Hough, Wei Huang, Sarah J. Lindsay, Daniel S. Malawsky, Davide Bonfanti, Dan Mason, Deborah Plowman, Michael A. Quail, Susan M. Ring, Gemma Shireby, Sara Widaa, Emla Fitzsimons, Vivek Iyer, David Bann, Nicholas J. Timpson, John Wright, Matthew E. Hurles, Hilary C. Martin

**Affiliations:** 1 Human Genetics, Wellcome Sanger Institute, Hinxton, England, CB10 1SA, UK; 2 Population Health Sciences, University of Bristol Medical School, Bristol, England, BS8 2BN, UK; 3 Bradford Institute for Health Research, Bradford Royal Infirmary, Bradford, England, BD9 6RJ, UK; 4 Sequencing R&D, Wellcome Sanger Institute, Hinxton, England, CB10 1SA, UK; 5 MRC Integrative Epidemiology Unit, University of Bristol, Bristol, England, BS8 2BN, UK; 6 Centre for Longitudinal Studies, University College London Institute of Education, London, England, WC1H 0NU, UK

**Keywords:** ALSPAC, MCS, BiB, WES, EGA

## Abstract

Birth cohort studies involve repeated surveys of large numbers of individuals from birth and throughout their lives. They collect information useful for a wide range of life course research domains, and biological samples which can be used to derive data from an increasing collection of omic technologies. This rich source of longitudinal data, when combined with genomic data, offers the scientific community valuable insights ranging from population genetics to applications across the social sciences.

Here we present quality-controlled whole exome sequencing data from three UK birth cohorts: the Avon Longitudinal Study of Parents and Children (8,436 children and 3,215 parents), the Millenium Cohort Study (7,667 children and 6,925 parents) and Born in Bradford (8,784 children and 2,875 parents). The overall objective of this coordinated effort is to make the resulting high-quality data widely accessible to the global research community in a timely manner. We describe how the datasets were generated and subjected to quality control at the sample, variant and genotype level. We then present some preliminary analyses to illustrate the quality of the datasets and probe potential sources of bias. We introduce measures of ultra-rare variant burden to the variables available for researchers working on these cohorts, and show that the exome-wide burden of deleterious protein-truncating variants, *S*_het_ burden, is associated with educational attainment and cognitive test scores. The whole exome sequence data from these birth cohorts (CRAM & VCF files) are available through the European Genome-Phenome Archive, and here we provide guidance for their use.

## Introduction

While exome sequence (ES) data for the UK Biobank ( Wang *et al.*, 2021) and other adult cohorts is catalysing the discovery of many rare variants and genes underlying late-onset complex diseases, there is a dearth of similar data from cohorts consisting of younger individuals from the general population. Furthermore, many cohorts with ES data are lacking longitudinal phenotype information. To enable research into how rare protein-coding variants impact phenotypes from infancy through to adolescence or early adulthood, we have recently carried out ES on three British birth cohorts at the Wellcome Sanger Institute (WSI). These are the Avon Longitudinal Study of Parents and Children (ALSPAC), the Millennium Cohort Study (MCS) and Born in Bradford (BiB). This effort seeks to enhance these internationally recognised long-term longitudinal studies, which, together, capture much of the UK’s diversity in terms of ethnicity, geography and socioeconomic indicators.

ALSPAC recruited 14,833 women based in Avon, UK who were pregnant during 1991 and 1992. This resulted in a total of 14,901 children from 15,447 pregnancies of predominantly white ethnicity (>95%). There has been engagement from 12,113 of the partners, with 3,807 currently enrolled. BiB is focused on the city of Bradford in the north of England, and recruited the mothers of 13,858 babies between 2007 and 2011, of whom ~41% self-report as white British and ~59% as other ethnicities, predominantly Pakistani. MCS is a UK-wide nationally representative cohort that recruited 18,818 children born between 2000 and 2002, intentionally over-sampling areas with high child poverty, large ethnic minority populations, and smaller UK nations (Wales, Scotland and Northern Ireland). The participants in these cohorts have been richly characterised over decades and each study has large archives of biosamples and data collected over different domains and dimensions ( Connelly & Platt, 2014; Joshi & Fitzsimons, 2016; Major-Smith *et al.*, 2022; McEachan *et al.*, 2024; Northstone *et al.*, 2019; Northstone *et al.*, 2023; Wright *et al.*, 2013). These studies also have a strong tradition of engagement with participants ( Calderwood *et al.*, 2015; Ochieng *et al.*, 2021) and the scientific community to enable high quality research. All adhere to the FAIR principles of data sharing (findability, accessibility, interoperability, and reusability).

Genotype chip data available on these cohorts have already been used to study the contribution of common genetic variants to phenotypes ranging from childhood obesity ( Bond *et al.*, 2022; Wright *et al.*, 2024) to parental nurturing behaviours ( Wertz *et al.*, 2023) and anxiety and depression ( Dennison *et al.*, 2024). The cohorts will be substantially enhanced by these ES data, which will additionally allow investigation of the role of rare protein-coding variants in a range of phenotypes. Extending the sequencing to first-degree relatives provides new opportunities to gain insight into *de novo* mutations ( Veltman & Brunner, 2012) and to tease apart direct genetic effects from indirect genetic effects plus confounders ( Davies *et al.*, 2024).

We aim to make these ES data accessible to researchers across a variety of disciplines. To that end, we have explained core concepts behind ES and its utility in Box 1 and given a high-level introduction to the generation and processing of exome data in Box 2. Additionally, we have indicated technical terms relating to ES and the quality control (QC) of ES data in red and described them in a Glossary (see Extended Data ( Koko *et al.*, 2024)). We have also given the background/motivation for each QC step at the top of each sub-section in the Methods.

**Box 1. Utility of exome sequencing**

The initial sequencing of the human genome ( Lander *et al.*, 2001) paved the way to genetic association studies that allow the identification of DNA variants affecting a broad range of human traits and diseases ( Auer & Lettre, 2015; Liu & Guo, 2016; Uffelmann *et al.*, 2021). These studies have predominantly used data from so-called “genotyping arrays” which allow one to query simultaneously several hundred thousand DNA variants that are known to be common in the population. Starting ~2008, the development of new technologies drastically reduced the cost of DNA sequencing, such that it became possible to sequence whole human genomes (or subsets thereof) much faster and more cheaply than before. The subsequent sequencing of DNA from large numbers of individuals has given us considerable insights into the role of rare genetic variants in human health and disease ( Belkadi *et al.*, 2016; Deciphering Developmental Disorders Study, 2015; Doan *et al.*, 2016; Ganna *et al.*, 2016; Gardner *et al.*, 2021; Marouli *et al.*, 2017). In particular, association studies focusing on the exome - which consists of all protein-coding regions within the genome - are a valuable tool to understand the relation of rare variants to various human traits ( Asimit & Zeggini, 2010; Chen *et al.*, 2022; Lee *et al.*, 2014; Park *et al.*, 2021; Povysil *et al.*, 2019; Wang *et al.*, 2021; Zuk *et al.*, 2014).

The human exome accounts for less than 2% of the genome, yet contains ~85% of known disease causing variants ( van Dijk *et al.*, 2014). Exome sequencing (ES) produces targeted DNA sequence data on the exome which can then allow focused analysis of the contribution of exonic genetic variation to phenotypic variation. In particular, it enables the direct assessment of protein-altering variants, the functional consequences of which are more readily interpretable than non-coding variants. This can provide information useful for the better understanding of potential mechanistic pathways between genetic variation and phenotypic variation. This can open up the possibility of gaining more direct therapeutic insights from genetic analysis ( Duffy *et al.*, 2024; Witkiewicz *et al.*, 2015). Using ES alone or in combination with other data sources has been shown to be a valuable resource, such as with rare-variant association testing ( Athieniti & Spyrou, 2022).

ES data can have broad and substantial utility for many researchers when coupled with detailed health and life course data measured on the same individuals. Since birth cohorts are very richly phenotyped and are widely studied, they provide a natural target for the collection of exome sequence data at scale. The data collected within each presented birth cohort is multidisciplinary, covering a wide variety of characteristics such as physical and mental health, child development, socio-economic factors and area/location-based characteristics. There is a large variety of overlapping phenotype data across ALSPAC, BiB and MCS. This overlap includes data such as anthropometrics (e.g., weight, height, BMI), socioeconomic indicators (e.g., parental and own education), behaviours (e.g., physical activity measured using accelerometers, diet, smoking, alcohol intake), cognitive and behavioural assessments (e.g., Strengths and Difficulties questionnaire, Denver Developmental Screening test), well-being measures (e.g., Warwick Edinburgh Mental Well-being scale), measures of vision, hearing, speech, medication usage and allergy history. The methods for data collection within these studies include combinations of questionnaires, face-to-face examinations at multiple time points, linkage to health and education records, and molecular phenotyping (e.g. genetic and metabolomic data). The phenotype data will grow richer as the study participants age. Combined with sequence data, these datasets therefore provide an opportunity to investigate the influence of rare damaging variants on a wide variety of phenotypes; standard genotyping arrays as used previously in these cohorts typically do not capture such variants, or do so with considerable limitations ( Sun *et al.*, 2021; Weedon *et al.*, 2021).

As these studies have strong relationships with their participants, they are well placed for recall-by genotype studies that could carry out follow-up work and functional analyses on individuals with interesting genotypes such as rare complete gene ‘knockouts’ ( MacArthur *et al.*, 2012; McGregor *et al.*, 2020; Minikel *et al.*, 2020; Narasimhan *et al.*, 2016; Saleheen *et al.*, 2017). The provision of family data within these cohorts allows investigation of cross-generation effects. For example, one can use data from duos (mother and child) within these cohorts to investigate the early development of the child ( Warrington *et al.*, 2019) or do trio-based analyses (i.e. analyses leveraging genetic data from biological mother, biological father and child) to detect *de novo* mutations ( Veltman & Brunner, 2012) or to disentangle direct genetic effects from genetic nurture or confounders ( Demange *et al.*, 2022; Young *et al.*, 2022).

**Box 2. Generating and processing exome sequence data**

Exome sequencing involves two main stages. In the first stage, exome capture, the DNA is fragmented, then fragments containing exonic sequences are selectively captured by molecular baits. The second stage involves the sequencing itself, in which the sequence of the captured DNA fragments is determined, producing ‘sequencing reads’, strings of adjacent molecular ‘letters’ in the DNA that are typically between 75 and 150 bases in length.

The exome sequence data is then processed as follows. The sequence reads are first aligned (‘mapped’) to a pre-defined reference sequence (the human reference genome) to determine the locations from which they originated. This process needs to account for several factors including naturally occurring variations between individuals, errors generated by the sequencing technology and the fact that the reads are short (a few hundred bases each) such that some cannot be mapped uniquely to the reference genome, particularly because of its repetitive nature. Various QC measures are used to ensure the quality of sequence data and to exclude contaminated samples. The differences between the exome sequence reads and the reference sequence are then examined in a process called ‘variant calling’, to determine the places where that individual’s DNA differs from the human reference genome. The variant calling process attempts to distinguish real variants from errors that are due to sequencing artefacts or mapping problems. It is necessary to undertake QC on the variant calls before conducting any analyses. The goal of QC is to ensure that the data is high quality, maximising the sensitivity so that as many true variants/genotypes are included as possible, but also maximising specificity so the number of false positive variants/genotypes is minimised.

## Methods

### Samples and consent

The methods for sample collection and profile of the ALSPAC cohort have been detailed previously ( Boyd *et al.*, 2013; Fraser *et al.*, 2013; Northstone *et al.*, 2019; Northstone *et al.*, 2023). Ethical approval for the study was obtained from the ALSPAC Ethics and Law Committee and the Local Research Ethics Committees and consent for biological samples has been collected in accordance with the Human Tissue Act (2004). Informed consent for the use of data collected via questionnaires and clinics was obtained from participants following the recommendations of the ALSPAC Ethics and Law Committee at the time. Children were invited to give assent where appropriate. Study participants have the right to withdraw their consent for elements of the study or from the study entirely at any time.

MCS is described in Connelly and Platt (2014), and Joshi and Fitzsimons (2016). The collection of DNA samples from MCS has been previously described ( Fitzsimons *et al.*, 2021). Ethical approval for the sixth sweep - which included the collection of saliva samples from children and biological resident parents - was obtained from London-Central REC (MREC; 13/LO/1786); informed consent was obtained from parents and children, including for biological samples in accordance with the Human Tissue Act (2004).

Recruitment and follow-up of BiB is described in McEachan *et al.* (2024), Shire *et al.* (2024) and Wright *et al.* (2013). Ethics approval for survey and clinic data collection, health record linkage, sample collection and follow-up was obtained from the National Health Service Health Research Authority Yorkshire and the Humber (Bradford Leeds) Research Ethics Committee. Consent for recruitment of the mother and baby to BiB was obtained from mothers upon attendance at a routine antenatal clinic appointment at the city’s main maternity unit, where the mothers were invited to complete an interviewer-administered questionnaire, and maternal and cord blood samples were taken.

The age at which biological samples were collected varied across cohorts. Whereas BiB collected umbilical cord blood at birth (< 500 children were sampled later), for this project, around one third of ALSPAC child samples were taken from those collected at birth and two thirds from around the age of 7 years; Saliva sampling for MCS was performed around age 14.

Extensive details of all available data and access regulations are available on the respective study websites (see Data Availability).

### Exome sequencing

For the exome sequencing, we considered samples from all children in these cohorts with sufficient DNA for library preparation, as well as from the parents of those for whom sufficient DNA was available from both mother and father. Around 2,000 BiB mothers of Pakistani origin have been previously exome sequenced at the WSI as part of a different project ( Narasimhan *et al.*, 2016). Similarly, approximately 3,000 ALSPAC children were previously exome sequenced at the Broad Institute for a different study ( Lam *et al.*, 2021; Wade *et al.*, 2021). If sufficient DNA remained, these same individuals were re-sequenced as part of the current effort in order to minimise batch effects.

DNA for 12,374 participants in ALSPAC (8,605 children and 3,389 of their parents) and 12,006 participants in BiB (9,041 children and 2,965 of their parents) were sent to the WSI from Bristol Bioresource Laboratories. Similarly, DNA samples for 15,240 participants in MCS (7,807 children and 6,975 of their parents) were sent to the WSI from University College London.

Each of the three cohorts was sequenced and processed separately at the WSI, following similar protocols. Samples were quantified upon arrival using the fluorescence-based Accuclear Ultra High sensitivity dsDNA kit and genotyped with a Fluidigm 192.24 chip which utilises twenty-two autosomal SNPs and two SNPs on the sex chromosomes to check for sample swaps. These were preliminary checks and were followed by detailed checks after variant and genotype QC, leveraging pedigree and sex information, as we explain later. Afterwards, 200 ng of genomic DNA was fragmented to an average size of ~ 150bp using LE220Rsc Ultrasonicator (Covaris Inc, MA, US), then purified and used to prepare exome sequencing libraries using NEBNext Ultra II DNA Library preparation Kit (New England Biolabs, MA, US). Next, 500 ng of pooled material was taken forward for hybridization, capture and enrichment using Twist capture baits (Twist Bioscience, CA, US), specifically the custom exome capture targets “Core exome plus Broad panel” (design ID: NGSTECustom_0001418). Libraries were sequenced on Illumina NovaSeq (Illumina, CA, US) S4 flow cells (100bp Paired-End reads) using version 1.5 reagents, with 112 samples per lane for ALSPAC, and 96 per lane for MCS and BiB. Reads were aligned to GRCh38 (including alt, decoy, and HLA contigs; 3,366 contigs) with BWA-MEM version 0.7.17 ( Li, 2013). We achieved an average on-target depth of ~62X for ALSPAC, ~69X for MCS and ~74X for BiB (Extended Data Figure 1).

### Outline of quality control

**Figure 1.**
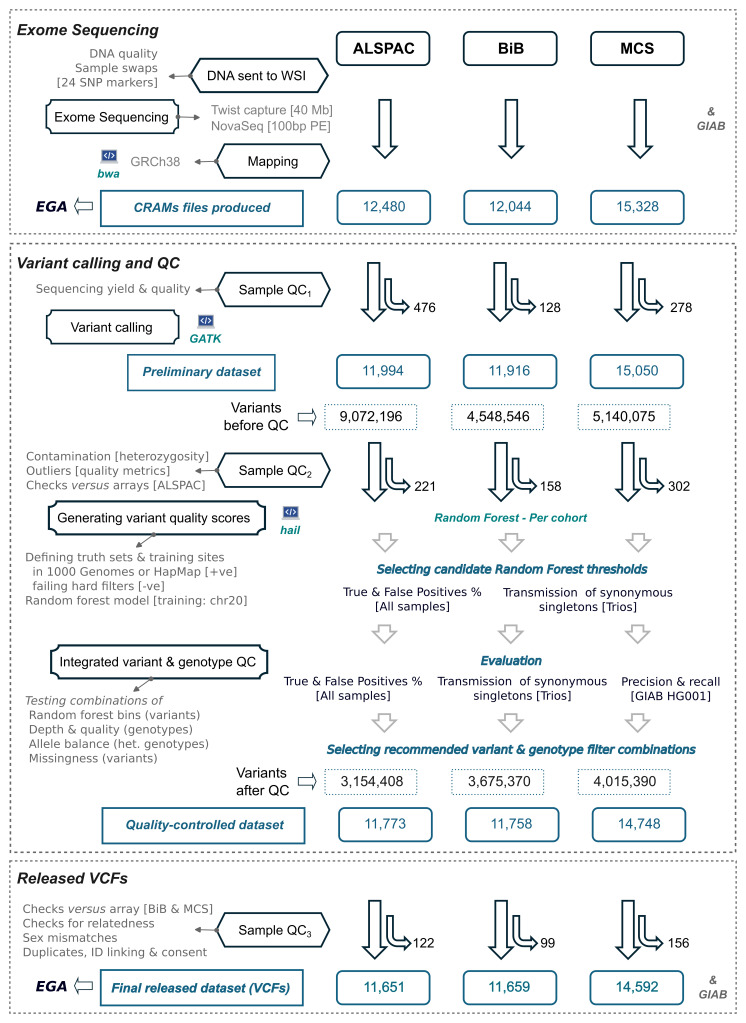
Overview of quality control steps and the sample size remaining at each step. The sample sizes shown do not include five Genome In a Bottle (GIAB) samples sequenced along each cohort. Note that the sample QC was performed in several stages. For ALSPAC, the checks for sample swaps against array data were performed on the preliminary dataset after variant calling (Sample QC _2_) while the other checks were performed in stage 3. ALSPAC had a disproportionately large number of variants before QC (but not after QC) due to a sequencing artefact that caused excess C>A mutations (see Box 3), which mostly failed random forest filtering (see the weight of the random forest feature ‘Is_CA’ in Table 2) or hard filters (see the cumulative ‘False Positives %’ in ALSPAC SNVs in Figure 3). The raw sequencing data (CRAM files) and the final VCFs were uploaded to the European Genome-Phenome Archive (EGA).

The three cohorts were processed separately, following similar protocols. The process of QC involved several stages ( Figure 1), which we outline briefly here before describing the details:

• Sample QC:
  ◦ Before variant calling: Samples were removed if they failed one or more filters based on quality of base-calls after sequencing, or quality of the CRAM files of aligned reads. The remainder then underwent variant calling.
  ◦ After variant calling: We assigned individuals to populations using principal component analysis (PCA), then identified and removed individuals who were outliers on one or more variant-based metrics within each of the populations. We compared the exome data to genotyping array data from the same samples and removed samples that did not match as expected, since these could be sample mix-ups. The samples were also checked for unexpected relatedness; samples showing conflicts between reported and inferred relatedness were removed. This sample QC was split in two separate steps, before and after variant and genotype QC, as detailed in the coming sections.

• Integrated variant and genotype QC:
  ◦ Variant QC: We removed candidate variants which may not be real, instead being artefacts or mapping errors, using a trained random forest model to distinguish likely true positives from likely false positives.
  ◦ Genotype QC: We removed low-quality individual genotype calls from the dataset. This was done in conjunction with variant QC, as we will explain below.

### QC of sequence data before variant calling

Background: *After sequencing and alignment to the reference genome, the ES data are first examined for general features of poor quality. This includes poor quality of called bases and sequence reads, low read depth or contamination. If samples do not pass these initial QC steps, they are unlikely to produce good-quality data so they are excluded.*

Prior to variant calling, some samples were removed based on various metrics measured in the CRAM files. The Q score (Phred quality score) ( Illumina, 2011) of each CRAM file was checked and CRAM files with a Q30 (corresponding to 99.9% base calling accuracy) of < 75 were rejected. In addition, checks were conducted based on sequencing yield, GC fraction, pulldown percentage, and on-target depth. Samples failing any of these checks were removed ( Figure 1).

### Variant calling (SNVs and indels)

Background: *Single nucleotide variants (SNVs) and small insertions/deletions (indels) are the most common type of genetic variation, and are ascertained from the raw ES data to allow downstream association analyses with phenotypes. This process tries to distinguish real variants from errors that are due to sequencing artefacts or mapping problems, by assigning several quality scores to the variants (averaged across all samples) and genotypes (for each individual sample). The variant calling process is followed by additional sample, variant and genotype QC described below, which improves upon this further by eliminating samples that are outliers, and variants and genotypes that are likely false positives.*

Single nucleotide variant (SNV) and small insertions/deletion (indels) calling was conducted with GATK HaplotypeCaller, GenomicsDBImport and GenotypeGVCFs (GATK version 4.2.4.0 for ALSPAC, v4.2.4.1 for MCS and v4.3.0.0 for BiB) following GATK best practices ( Van der Auwera & O’Connor, 2020).

Sample, variant and genotype QC were then conducted as described below, using Hail v0.2.97 for ALSPAC and v0.2.105 for MCS and BiB (see Software Availability). Sample and variant QC were based on methods used by the Genome Aggregation Database (gnomAD), which were described in ( Karczewski *et al.*, 2020).

### Sample QC

***Checks for contamination***

Background: *In addition to removing samples with low sequence data quality (‘QC of sequence data before variant calling’), we also checked for an excess of heterozygous genotypes which suggests possible contamination.*

Using VerifyBamID v2 ( Jun *et al.*, 2012), we flagged three samples in MCS and two samples in BiB that had marginally higher free-mix scores than the default threshold of 0.05 (range: 0.05 - 0.055); since these samples were not far off from the remaining samples when we visualised the distribution of the scores, we did not exclude them. In ALSPAC, we flagged 19 samples that had a score > 0.05 [range: 0.05 - 0.5], which we then removed from the downstream analysis. The conservative choice to remove failing samples in ALSPAC was partially motivated by the existence of sequencing artefacts in some sequencing batches (described below); we felt that reliable bioinformatic identification of these artefacts would benefit from removing other potential sources of low quality variant calls.

***Population prediction by Principal Component Analysis (PCA)***

Background: *People with different genetic ancestries vary in the number of genetic variants in their genomes, as well as on other metrics such as the ratio of heterozygous to homozygous genotypes. Examining these sample-level metrics is a useful QC step, but the metrics only make sense when compared between individuals with similar genetic ancestries. Thus, before completing the sample QC, we need to assign individuals to genetic ancestry groups, which we do by combining our own data with data from reference samples from the 1,000 Genomes Project.*

**Figure 2.**
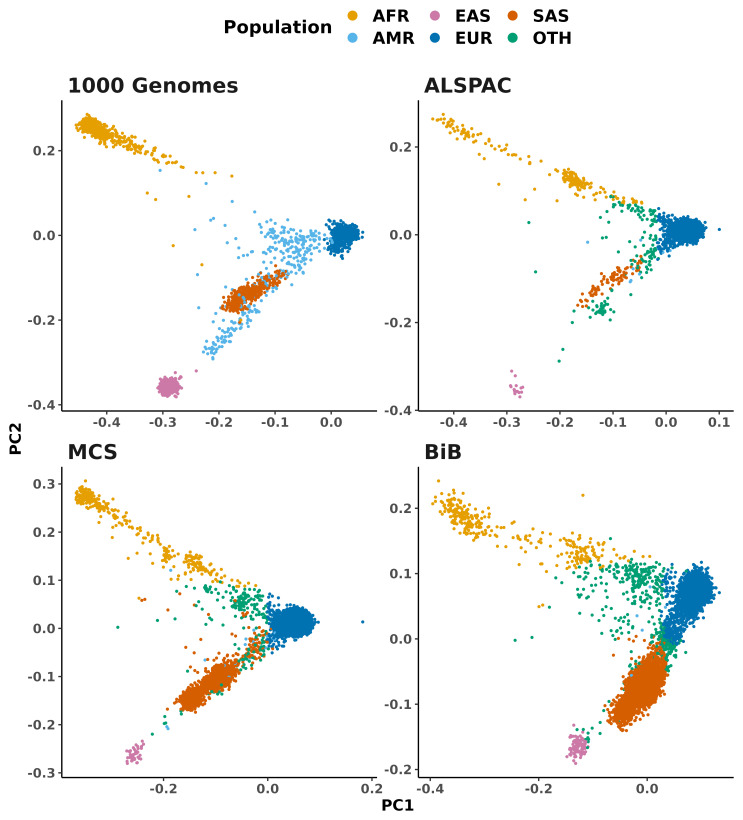
PCA plot showing the continental ancestry groups for samples from the 1000 Genomes Project and UK birth cohorts. The birth cohort samples are coloured by continental population labels (‘super-populations’) inferred from their similarity to the 1000 Genomes samples. Top-left: The 1000 Genomes samples. Top-right: Avon Longitudinal Study of Parents and Children (ALSPAC). Bottom-left: Millennium Cohort Study (MCS). Bottom-right: Born in Bradford study (BiB). Note that the PCA was performed separately for each cohort (merged with 1000 Genomes, as described in Methods), therefore the axes and scale are not identical between cohorts. AFR: African, AMR: Admixed American (sometimes referred to as Hispanic-Latin American), EAS: East Asian, EUR: European, SAS: South Asian, OTH: other (ie. could not be confidently inferred).

Before assigning people to populations based on their genetics, the data were filtered to only autosomal variants, and variants in linkage disequilibrium were pruned using Hail’s ld_prune function with an r ^2^ of 0.2. The ES data from each cohort were merged with samples from 1,000 Genomes from known populations, and variants present in both the birth cohort data and the 1,000 Genomes data were retained. Further filtering was performed to remove variants with low call rate (< 0.99), low allele frequency (< 0.05), low Hardy-Weinberg equilibrium p-value (< 1x10 ^-5^), variants in long range linkage disequilibrium regions and palindromic SNVs. PCA was run using Hail’s hwe_normalized_pca function, and gnomAD’s assign_population_pcs function was used to predict which continental-level ancestry group each birth cohort sample belongs to based on the PCs. Figure 2 shows the first two principal components of this PCA space to highlight the inferred genetic ancestry labels for birth cohort samples in relation to the 1,000 Genomes samples. Table 1 gives the number of samples inferred to come from each continental-level ancestry group. 

**Table 1.** Number of samples per cohort in each superpopulation before and after QC.

Dataset	Included in variant calling			Final release		
Cohort	ALSPAC	BiB	MCS	ALSPAC	BiB	MCS
African (afr)	200	311	340	188	305	326
Admixed American (amr)	10	6	22	8	6	19
East Asian (eas)	17	94	42	16	94	40
European (eur)	11,537	5,174	12,847	11,219	5,034	12,560
South Asian (sas)	84	5,896	1,559	83	5,791	1,441
Other (oth)	146	435	240	137	429	206
All	11,994	11,916	15,050	11,651	11,659	14,592

**Table 2.** The features used in the random forest used for variant QC, and their relative importance. Features based on gnomAD methods QC are indicated by asterisks.

Feature	Description	Importance		
		ALSPAC	MCS	BiB
QD*	Quality normalised by depth	0.4353	0.491	0.4387
meanHetAB	Mean fraction of reads carrying the alternate allele (allele balance) at heterozygous genotypes	0.3109	0.321	0.2593
is_CA	Is a C>A/G>T SNV	0.1808	0.001	Not used
SOR*	Strand odds ratio	0.0617	0.047	0.1008
variant_type*	SNV/indel/multiallelic SNV/multiallelic indel/multiallelic mixed	0.0034	0.003	0.0138
ReadPosRankSum*	Rank sum test for relative positioning of REF versus ALT alleles within reads	0.0027	0.013	0.001
was_split	Split multiallelic site	0.0013	0.017	0.0504
has_star*	Alleles at this site include an overlapping deletion	0.0011	0.013	0.0179
n_alt_alleles*	Number of alt alleles at a site	0.0008	0.002	0.0054
MQ	Mapping quality	0.0013	0.086	0.1021
MQRankSum*	Rank sum test for mapping qualities of REF versus ALT reads	0.0002	0.005	0.0076
allele_type*	SNV/insertion/deletion	0.0002	0.002	0.0026
was_mixed*	Multiallelic site containing SNV(s) and indel(s)	1.4e-5	0.0001	0.0005

***Removal of outlier samples stratified by superpopulation***

Background: *Samples were compared to others within the same superpopulation on several metrics to find outliers who may have had poor sequencing or DNA quality. In theory, if a large number of individuals from the same sequencing batch across different ancestry groups are flagged as outliers, this check can help pick up egregious batch effects within a dataset, before more complex QC is applied.*

For the purpose of determining outlier samples, prior to calculating the sample quality metrics, the exome variant calls were pre-filtered to set the genotypes with total allele depth (DP) < 20, genotype quality (GQ) < 20 or variant allele fraction (VAF) < 0.25 (heterozygous calls) to missing. Hail’s sample_qc function was then run and the following metrics (defined in the Glossary; see Extended Data) were calculated per sample:

- Number of SNVs.
- Number of transitions.
- Number of transversions.
- Transition/transversion (Ti/Tv) ratio.
- Number of deletions.
- Number of insertions.
- Insertion/deletion ratio.
- Heterozygote/homozygote ratio.
- Heterozygosity rate.

The output was stratified by inferred superpopulation for each cohort, and a sample was deemed to fail sample QC if it fell outside of the median +/-4 medium absolute deviations (MAD) for any one metric when compared to samples from the same cohort and superpopulation.

We note that pre-filtering genotypes on DP, GQ and VAF applied only to the calculation of these sample metrics, and a formal genotype QC was performed later on (see below). Pre-filtering ensures that sample metrics are not skewed by low-quality genotypes that are likely to fail any reasonable QC strategy. This was particularly relevant in ALSPAC, in which one hundred and thirteen samples were found to have a roughly three-fold excess of SNVs prior to QC (see Box 3); these, and other samples with sequencing artefacts, initially skewed the median/MAD values for several metrics and resulted in a high number of other samples failing without truly being outliers. Table 1 shows the number of samples per cohort that were deemed to be outliers on at least one metric and thus failed sample QC.

**Box 3. Mutational spectrum and artefacts**

Background: *Based on the similarity of their biochemical structures, the four DNA building blocks (letters) can be divided into two groups: purines (A/G) and pyrimidines (C/T). Changes from one base to a structurally similar base are called transitions (A>G, G>A, C>T, T>C), whereas changes between the two groups are called transversions. Naturally occurring variations are more frequently seen between bases that are structurally similar, and transitions are (at least) twice to three times as frequent as transversions in exome sequence data. Sometimes systematic errors occur during the library preparation or sequencing (e.g. in specific batches or sequencing runs), which often appear as an excess of one of these types of changes.*

The mutation spectra and transition-transversion ratios were within the expected range for most samples and sequencing batches. A subset of ALSPAC and, to a lesser extent, some MCS samples, were found to be affected by a pervasive C>A artefact which proved to have occurred during library preparation and required correction (Extended Data Figure 2). This artefact is one of the commonly observed errors in ES and results from oxidation of Guanine to 8-Oxo-Guanine (OxoG) in pre-adaptor regions ( Costello *et al.*, 2013). In contrast to Guanine, which binds Cytosine during PCR, Oxo-Guanine can bind to Adenine as well (i.e. introducing A instead of C), which manifests as an excess of C>A/G>T changes in variant calling. Since sequencing artefacts usually have particular patterns, it is possible to remove them bioinformatically ( Costello *et al.*, 2013). We carried out the variant and genotype QC described below to remove this artefact from the dataset. After completing the sequencing of ALSPAC, part way through the sequencing of the MCS cohort, and before the sequencing of the BiB cohort, we altered the library preparation protocol to elute both pre- and post-capture librarie(s) in elution buffer rather than water, which removed this artefact.

### Generating quality scores for variant QC

Background: *The goal of variant QC is to remove variants that are likely to be artefacts of sequencing or mapping problems, while retaining as many true variants as possible. For this purpose, machine learning models, trained on a number of different metrics in well-defined truth sets, are used to distinguish likely true positives from likely false positive variants. The performance of these models is then evaluated to choose appropriate cut-offs that maximise the true positives and minimise the number of false positives.*

Before QC, there were 9,072,176 variants in ALSPAC, 5,140,075 in MCS, and 4,548,546 in BiB. We trained a random forest model on a subset of variants likely to be clear true or false positives based on their occurrence in known truth sets or their quality metrics, and ran it on the remaining variants to assign a score representing the quality of the variant. Since the quality of the dataset is determined not only by which variants are included but also the quality of the genotypes retained at those variants, the final QC thresholds were decided by considering different combinations of random forest thresholds with various genotype filters based on depth (DP), quality (GQ) and allele fraction (VAF). We started by nominating a range of random forest thresholds then combined these with selected genotype filters. Next, we applied selected thresholds for these random forest scores combined with selected genotype filters (i.e. we tested a grid of random forest, DP, GQ, and VAF thresholds), and at the same time considered different missingness thresholds, to determine appropriate filtering thresholds based on precision, recall, and percentage of true positives and false positives. The details of this QC are presented in the following sections.

***Preparation of truth set and negative training set***

Background: *Genetic variants commonly detected in large-scale population studies using different technologies or in well-validated experiments are considered to be high-confidence variants; therefore, these are typically used as truth sets when training machine learning models for quality control purposes. Defining a set of variants that are known to be errors or artefacts is less straightforward and usually dependent on the context. Most of the time, variants failing several lenient thresholds for quality metrics are considered to be of low-confidence*.

Variants present in the following high-quality datasets were identified in our data. These were deemed to be true positive variants:

- High confidence SNVs discovered in 1000 Genomes ( Auton *et al.*, 2015).
- SNVs present on the Illumina Omni 2.5 genotyping array and found in 1000 Genomes.
- High-confidence indels present in the Mills and Devine data ( Mills *et al.*, 2006).
- SNVs and indels from the HapMap3 project ( International HapMap 3 Consortium *et al.*, 2010).

We defined the following number of true positive variants: 650,027 SNVs and 17,329 indels in ALSPAC, 673,480 SNVs and 17,579 indels in MCS, and 674,752 SNVs and 17,621 indels in BiB.

Variants failing one or more of the following hard filters were deemed to be erroneously called false positive variants, and thus were used to construct a training set for negative sites: Quality by Depth (QD) score < 2; FisherStrand (FS) score > 60; Mapping Quality (MQ) score < 30 (refer to the Glossary for metric definitions; see Extended Data).

The number of false positives per cohort was as follows: 4,872,299 SNVS & 96,192 indels in ALSPAC, 430,990 SNVs & 105,345 indels MCS, and 198,711 SNVs & 84,914 indels in BiB. The very large number of false positives in ALSPAC reflects the ‘C>A’ artefact.

***Training and applying the random forest.*** Background: *A random forest model is a machine learning algorithm which uses decision trees to classify points (in this context, genetic variants) into two groups (in this context, likely true variants versus likely erroneous variants). It starts by considering several input values or measurements (e.g., measures of variant quality, usually called features) in a pre-defined training set in which the points in the two groups are already known (in our case, ‘correct’ versus ‘erroneous’ variants). It learns an optimal combination of input values (decision tree) to distinguish between the groups (likely true positives and false positives) in other datasets of similar nature.*

For each cohort, we trained a random forest model on a smaller chromosome (chr20) to distinguish true versus false positive variants, applied it to all variants, evaluated several performance metrics, and from this selected QC thresholds which we then applied to all chromosomes. The features used in the random forest and their relative importance are shown in Table 2. The feature choice was largely based on the set of features used by gnomAD for their QC ( Karczewski *et al.*, 2020). We added several metrics as additional features, namely:

- the mean heterozygous allele balance (meanHetAB), i.e. the average fraction of reads carrying the alternate allele at a heterozygous genotype
- the average mapping quality of all the reads on which a given variant call is based (MQ)
- an indicator for whether the variant was in a split multi-allelic site (was_split)

In ALSPAC and MCS, a feature “ is_CA” was also added to indicate if an SNV is a C>A (or G>T) substitution to attempt to identify and subsequently remove the excess C>A/G>T substitutions in these datasets. This feature was not used in BiB as it was sequenced after a change in library preparation protocol to eliminate this artefact.

As Table 2 indicates, quality by depth was the most important feature, as previously shown in gnomAD ( Karczewski *et al.*, 2020). ‘Is_CA’ had a high relative importance in ALSPAC and low importance in MCS.

***Random forest evaluation***

Background: *The random forest assigns a score to each variant which reflects how likely it is to be a true variant. We needed to decide which threshold to set in order to retain a variant. This choice of threshold will impact the sensitivity and specificity of the final variant callset.*

**Figure 3.**
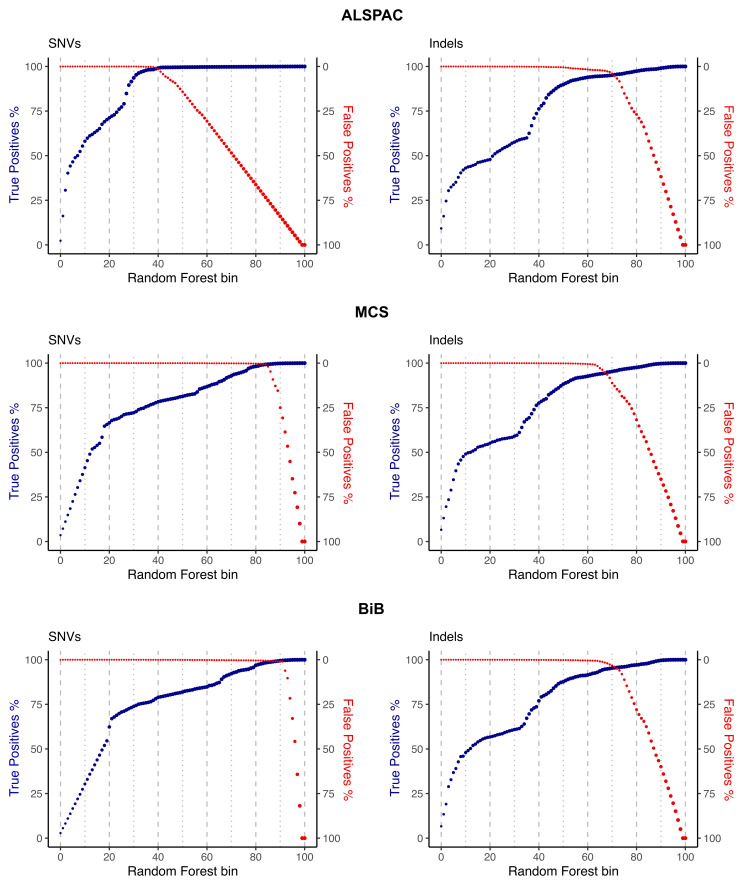
True and false positive rates plotted against random forest bins for single nucleotide variants (SNVs) and insertions-deletions (indels). The plots are cumulative, such that the y-axis depicts the overall indicated metric (percent of true or false positives identified) for SNVs or indels in bins lower than and including the bin indicated on the x-axis.

To determine a starting point for choosing filtering thresholds (described in the next section), variants were first ranked by random forest score and binned into a hundred groups. Next, we visually inspected three performance metrics: the cumulative number of true positive variants and the cumulative number of false positive variants per bin (for both SNVs and indels ( Figure 3)), and the ratio of the transmitted/untransmitted synonymous singletons (Extended Data Figure 3).

When looking at true/false positive variant counts, the goal is to find a range of QC thresholds that maximise the true positives and minimise the false positives. The transmitted/untransmitted ratio for synonymous singletons is a particularly useful metric that makes use of the trios and examines synonymous variants seen in only one parent in the dataset. If the QC is properly calibrated, we expect such variants to be transmitted to the child 50% of the time. Hence, we want to choose the QC thresholds so that this ratio is as close as possible to 1 while also optimising the other metrics outlined in the next section.

### Integrated variant and genotype QC

Background: *Whereas variant QC tries to distinguish real variants from artefacts, genotype QC tries to remove low-quality genotypes of individual samples at a given variant site. The goal is to help ensure any downstream work will be accurate and reliable.*

To decide on suitable hard filters on variants, combinations of random forest bins (i.e. a variant-level metric) were tested with various genotype quality metrics: DP, GQ and HetAB. Specifically, at variants passing a given random forest bin filter, genotypes were then set to missing if they had GQ, DP or HetAB (variant allele fraction) less than the specified threshold. For each potential combination of filters examined, various metrics were calculated:

- The percentage of true positive and false positive variants from the random forest annotation remaining.
- The transmitted/untransmitted synonymous singleton ratio.
- Precision and recall of variants found in the Genome in a Bottle (GIAB) sample GIAB12878/HG001 ( Zook *et al.*, 2016). This individual has been previously sequenced using multiple technologies and undergone very comprehensive variant calling. Hence, a very high quality variant call set with high sensitivity and specificity is available for this individual, and, after applying different filters to our data, we compared our dataset to this in order to determine the precision and recall.

**Figure 4.**
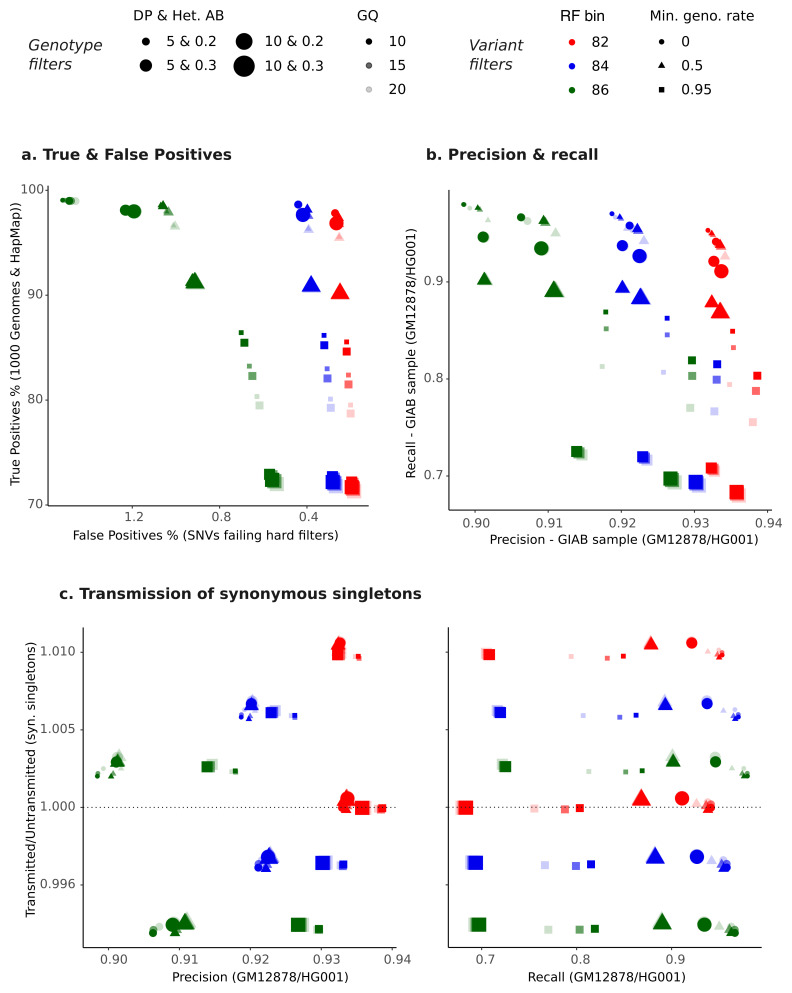
Integrated variant and genotype QC for SNVs in MCS. The plots show the following metrics obtained after applying different combinations of variant- and genotype-level filters: **a**) true versus false positive rate, **b**) recall versus precision for sites from the reference GIAB sample, and **c**) transmitted/untransmitted ratio for synonymous singletons versus precision (left) or versus recall (right). In each plot, the points show the value of the metric for a particular combination of filters. The colour indicates the random forest (RF) bin filter, the opacity of the point indicates the genotype quality (GQ) filter, the size of the point indicates the filter on a combination of genotype depth (DP) and heterozygous allele balance (Het. AB), and the shape of the point indicates the minimum genotyping rate (min. geno. rate) after applying genotype QC (i.e. missingness filter). For plots of true/false positives and precision/recall in all three cohorts, see Extended Data Figure 4 (SNVs) and Extended Data Figure 5 (indels).

We explored a range of random forest bins guided by the balance between true and false positives ( Figure 3) and transmitted/untransmitted ratios (Extended Data Figure 3). We also examined these outcomes after applying additional filtering on missingness (i.e. removing variants with genotyping rates < 50% and < 95%, after applying the genotype filters). This is exemplified in Figure 4 which shows the performance outcomes for three SNV random forest bins in MCS when combined with genotype and missingness filters. Examples of variant and genotype filter combinations for SNVs and indels all three cohorts are shown in Extended Data Figure 4 (SNVs precision/recall & true/false positives) and Extended Data Figure 5 (indels precision/recall & true/false positives).

After exploring these metrics, we settled on three combinations of relaxed, medium, and stringent filters for each cohort (Extended Data Table 1) that we thought provided reasonable balance between all metrics, with the relaxed filters slightly favouring recall of GIAB variants and the stringent set favouring precision. The definitions of filter combinations differed for each cohort since it became clear that the same filters performed differently in the various cohorts (Extended Data Table 2).

Stringent QC in ALSPAC substantially decreases the false positive rate compared to the medium QC, with a more modest decrease in true positive rate (Extended Data Table 2). Reassuringly, the mutation spectrum was very similar across all cohorts after this stringent QC (Extended Data Figure 2b). Extended Data Figure 6 shows that applying the stringent filters reduces the impact of the C>A artefact in the worst-affected samples. Thus, we chose to prioritise the stringent filters in ALSPAC, achieving the metrics shown in Table 3. More stringent filtering on either of RF bin, DP, and AB in MCS and BiB resulted in better balance of transmitted/untransmitted ratio, higher precision and lower false positives (but lower recall and lower true positives) as shown in Extended Data Figure 4 and Extended Data Figure 5. Stringent filters had similar performance metrics in MCS and BiB, and both were better than ALSPAC on most metrics ( Table 3).

**Table 3.** Summary of performance metrics for the three sets of stringent QC filters chosen. *This table shows QC metrics obtained after applying the indicated filters, which were the final set chosen for the main data release: precision/recall for the Genome In A Bottle sample GM12878/HG001, percentage of true positive and false positive calls from the random forest inputs remaining in each dataset, and transmitted/untransmitted ratio for synonymous singletons. These values were obtained after applying the recommended stringent filters shown in the table. For more information, see the following related tables: Extended Data Table 1 (definitions of tested filters), Extended Data Table 2 (performance metrics for SNVs) and Extended Data Table 3 (performance metrics for indels).*

Variants	SNVs			Indels		
Cohorts	ALSPAC	MCS	BiB	ALSPAC	MCS	BiB
**Recommended filters** (level applied)						
Random forest bin (variant)	36	82	84	56	58	58
Depth (genotype)	5	5	5	10	10	5
Genotype quality (genotype)	20	15	15	10	20	20
Heterozygous allele balance (genotype)	0.2	0.2	0.2	0.3	0.3	0.3
Genotyping rate (variant)	50%	50%	50%	50%	50%	50%
**Performance evaluation**						
Precision	0.912	0.933	0.933	0.802	0.796	0.779
Recall	0.916	0.948	0.951	0.524	0.612	0.707
True positives (%)	93.28	96.846	97.048	72.39	89.512	87.778
False positives (%)	0.10	0.254	0.462	0.48	0.344	0.250
Transmitted/untransmitted ratio	0.979	1.010	0.988	n/a	n/a	n/a

### Additional sample QC for sample swaps

***Checking sample identity in exome data against array data***

Background: *Cross-checking the exome sequence against existing genotyping array data for each individual allows us to see if the genotypes of variants detected by both technologies match as expected. If an individual does not match between the exome and array samples (allowing for some low level of genotyping error), we can check their genetic relationship with people in the exome or array data whom we predict to be first-degree relatives to try and resolve which sample is incorrect. The goal of this is to detect sample mix-ups and remove samples accordingly.*

ALSPAC samples (N=20,007) were genotyped previously using the Illumina HumanHap550 quad (children), Illumina Human660W (mothers) and Illumina CoreExome array (partners) ( Taylor *et al.*, 2018). MCS samples (N=21,181) were previously genotyped on the Global Screening Array (GSA) ( Fitzsimons *et al.*, 2021). BiB samples (N=19,293) were previously genotyped on the Illumina CoreExome array and GSA ( Arciero *et al.*, 2021; Bird *et al.*, 2019). Bcftools gtcheck ( Danecek *et al.*, 2021) was used to identify array-genotyped samples that match with each exome sample. A good match was defined by having a discordance score < 0.05 (i.e. number of discordant genotypes in a pair of samples divided by the total number of non-missing sites compared).

We removed exome samples that did not have a good match in the array data where a match was expected. When the best match was not the expected array sample, we looked at the genetic kinship to first-degree relatives and decided whether to remove the exome sample based on these scenarios: (i) When the exome sample did not have an expected first degree relative to confirm the genetically inferred relationship, the exome sample was removed; (ii) If the individual was expected to have first-degree relatives in the exome dataset but their inferred (genetic) relationship was not as expected, the exome sample was removed; (iii) When exome sample showed the expected genetic relatedness to first-degree relative(s) in the exome dataset, it was not removed (i.e. we assumed the mismatch was due to a sample mix-up in the array data); (iv) Samples for whom the best hit was the monozygotic twin of the expected sample were not removed.

Through these checks, the following numbers of samples were removed from the exome datasets: 105 from ALSPAC, 27 from MCS and 55 from BiB. Note that, for ALSPAC, this step was completed prior to the previous steps of variant and genotype QC described above, whereas for MCS and BiB, it was carried out after variant and genotype QC.

***Relatedness checks within families within the exome data***

Background: *If several related individuals are sequenced, checking the genetic relatedness against the reported family structure may pick up mis-labelled samples or sample swaps that were not identified in the previous checks e.g. because they were not genotyped or the sample mixup happened in both array and exome datasets. For example, a mismatch in expected versus observed genetic relatedness between only one sample and the remaining samples in a family suggests that the labelling of this sample may have been wrong and it can be removed. A sample swap within a family may also be revealed through these checks. Similar to the previous check, the goal of this step is to detect sample mix-ups and remove samples accordingly from the final released dataset*.

After excluding exomes that failed QC, the free-mix contamination check, or the checks against array data, and carrying out the variant and genotype QC described above, we further checked the relatedness within each exome-sequenced family (i.e. whether expected parent-offspring pairs were inferred to be parent-offspring genetically and whether expected sibling pairs were inferred to be siblings genetically).

We removed both the child and the parent from problematic pairs when there was no other family member to confirm their relationship, and we removed only the parent when the other parent showed expected relationship with the child. We removed 62 exomes from ALSPAC, 34 from MCS and 29 from BiB.

***Sex check***

Background: *If there is no existing array data for an individual, and no other first degree relatives in the exome dataset, we can at least compare the predicted sex with what is reported for an individual. If these do not match, it suggests a sample mix-up so samples are removed accordingly.*

For the inference of biological sex, the data were filtered to include only the samples that were not removed during other sample QC procedures, and to remove variants with an internal allele frequency of < 0.01 or a call rate of < 0.99. The sex of each sample was imputed using the inbreeding coefficient on the X chromosome (calculated using PLINK) and the total depth of coverage on the Y chromosome (calculated using bcftools). The two metrics were plotted and showed the two main expected clusters (males with inbreeding coefficients close to 1 and high coverage on chromosome Y, and females with both low inbreeding coefficients and low coverage on chromosome Y). Samples falling in these clusters were assigned the corresponding sex, which was then compared to the reported sex. The following numbers of discrepancies were identified and removed from the final cohorts: 3 for ALSPAC, 3 for MCS and 12 for BiB.

***Other removed samples***

Background: *Genetically similar (duplicate) samples that are neither from twins nor from individuals where two samples were (un)intentionally sequenced (ALSPAC contained duplicate samples for QC purposes) may reflect sample swaps. We examined the datasets to remove potential problematic duplicate samples and ensure all genetic duplicates in the final release are twins. We also removed any samples where the consent was withdrawn and those with linking errors (e.g., missing details on sample identifiers).*

Following QC, ALSPAC contained 71 pairs of duplicate samples (139 exome samples with one or more genetic duplicates in the cohort). Thirteen pairs (26 samples) were twins, and were included in the final release, whereas 58 pairs (113 samples) were not twins; Of these, 54 pairs (108 samples) were intentionally duplicated samples sequenced from the same individuals for QC purposes, and four pairs (eight samples) were from seven individuals with discordant family identifiers, suggesting sample swaps. We removed 60 samples from these 113 samples, as described here:

- For 53/54 pairs, we prioritised the sample with the higher average bait coverage, higher average depth at called variant sites (after applying stringent filters and including variants with less than 50% missingness), and higher genotyping rate (after applying the same filters), taking the sample that performed best in two (or all three) of these comparisons.
- The remaining pair of the 54 intentionally duplicated samples passing QC was also a genetic match to a third sample with a different individual identifier (combination of family and ‘qlet’ identifiers). None of these samples had array data or exome data from relatives to validate their identity, so all three were removed.
- Similarly, we removed four samples (two pairs of duplicates) from four individuals with discordant family identifiers that were not intentional duplicates.

BiB had 19 pairs of duplicate samples after QC, including 16 pairs of samples mapped to twins. The remaining three pairs of duplicates were samples from the same individuals sent for sequencing at two different time points; We removed the second sample in each pair for simplicity (i.e. removed three samples duplicated unintentionally).

After QC, we retained 23 pairs of duplicate samples in MCS - all from twins. Otherwise, there were no duplicate samples. We removed 8 samples due to withdrawn consent and 84 samples because they could not be confidently linked to a corresponding individual in MCS (e.g. missing information).

## Dataset validation

Background: *We performed several analyses to explore the quality of the exome data set after the described filters were applied. These analyses were aimed at spotting unwanted sources of variability as well as reproducing known phenotypic associations with selected traits. To perform these analyses, we annotated the variants with their expected consequence for the protein sequence (e.g. no change, shorter protein that will be degraded, a protein with a single altered amino-acid) and filtered them based on the frequency they are observed at in the birth cohorts and other population datasets. We then performed statistical analyses to test for significant associations between the burden of these variants and the traits measured in each cohort or to compare variant counts between samples whose DNA came from different sources*.

### Methods for data validation section

***Evaluating the effect of QC stringency on sample metrics***

**Figure 5.**
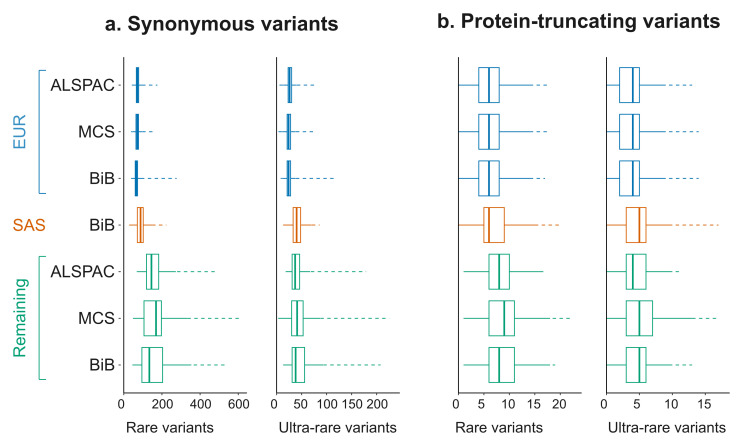
Rare and ultra-rare variant counts in the birth cohorts. The boxplots show the median and interquartile range (IQR) after applying the recommended filters shown in Table 3. Two (gnomAD) allele frequency thresholds are shown (rare: 0.1%; ultra-rare 0.003%). The whiskers show the range excluding outliers (solid lines) or including outliers (dashed lines). The largest sample groups in terms of predicted genetic ancestry (European (EUR) in the three cohorts and South Asian (SAS) in BiB) are plotted separately from the remaining samples.

We annotated the variant consequences on MANE transcripts along with gnomAD allele frequencies (genomes r3 and exomes r2), loss-of-function confidence (LOFTEE) and deleteriousness scores (CADD) using VEP 108 ( McLaren *et al.*, 2016; Rentzsch *et al.*, 2019). High-confidence PTVs were defined as those annotated as stop-gained (with a CADD score > 25), splice-donor, splice-acceptor, or a frameshift variant, and considered ‘high-confidence’ by LOFTEE ( Karczewski *et al.*, 2020). In all three cohorts, rare variants were defined as those with an allele frequency < 0.1% amongst (i) a set of unrelated probands from the birth cohort being studied (internal minor allele frequency), and (ii) gnomAD r3 genomes and gnomAD r2.1 exomes (external allele frequency) ( Karczewski *et al.*, 2020). Ultra-rare variants were defined as those with an internal allele frequency < 0.1% and an external allele frequency in gnomAD < 0.003% (approximately, less than 5 individuals in 76k gnomAD r3 genomes and less than 10 individuals in 141k gnomAD r2 exomes). When defining rare and ultra-rare variants in BiB, which had two main genetic ancestry groups ( Table 1), we added another frequency filter based on the maximum allele frequency in the European and South Asian genetic ancestry groups (i.e. allele frequency < 0.1% in both). The distribution of rare and ultra-rare protein-truncating and synonymous variants after applying the recommended filters is shown in Figure 5.

***Exome-wide rare variant burden associations***

Ultra-rare PTVs in loss-of-function intolerant genes were previously found to be associated with reduced cognitive ability and educational attainment ( Ganna *et al.*, 2016; Gardner *et al.*, 2022). We used *s*_het_ - a gene-level measure of the degree of negative selection against PTVs (“constraint”) ( Cassa *et al.*, 2017) - to construct an exome-wide measure of ultra-rare variant burden, and examined the association of this measure with various phenotypes related to cognitive ability. Gene-level *s*_het_ scores were obtained from Agarwal *et al.* (2023) and used to construct an exome-wide *S*_het_ burden score for each individual *i* as follows, following Gardner *et al.* (2022):


$$S_{het}\;burden_{\left[i,v\right]}=1-\prod_{g}\left(1-s_{het[i,v,g]}\right)$$

where *S*_het_ *burden*_[ *i,v*]_ indicates individual *i*’s *S*_het_ burden for variant class *v*, and *s*_*het* [ *i,v,g*]_ indicates the *s*_*het*_ score for gene *g* containing a qualifying variant in class *v* in individual *i*.

**Figure 6.**
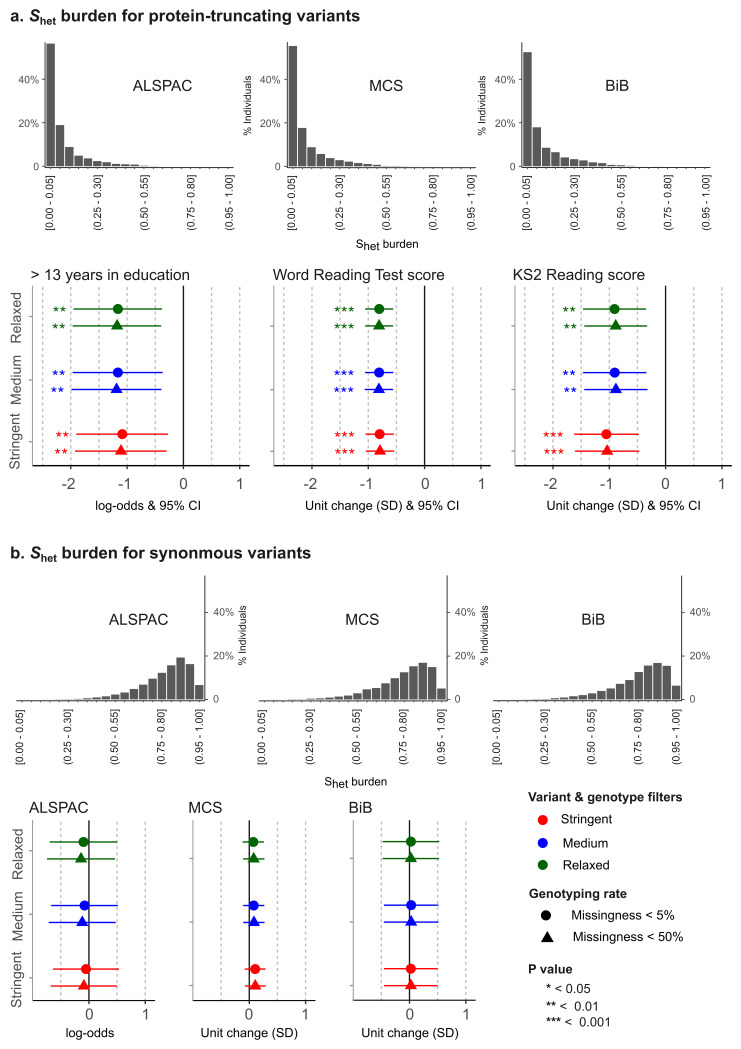
Association between S _het_ burden for ultra-rare protein-truncating and synonymous variants and education-related phenotypes. S _het_ burden is a measure of exome-wide ultra-rare variant burden (see the Methods for more details on S _het_ burden derivation). The distribution of S _het_ burden in individuals of predicted European genetic ancestry is shown in ( **a**) for protein-truncating variants and ( **b**) for synonymous variants. The plot also shows the association of these scores with educational/cognitive test scores. For each set of QC filters on the y axis, the x axis shows the log-odds of having more than 13 education-years (ALSPAC) and the change (standard deviations) in reading test scores (MCS and BiB) for an S _het_ burden of 1 (versus 0) calculated using protein-truncating variants ( **a**) and synonymous variants ( **b**).

We calculated this *S*_het_ burden score using high-confidence PTVs. Higher *S*_het_ burden for PTVs indicates that the individual has a relatively higher contribution from PTVs in genes that are under stronger negative selection in the general population. We also calculated *S*_het_ burden scores for synonymous variants to assess if any observed PTV associations are biassed by residual population or technical (sequencing) differences. When calculating these scores, we included only ultra-rare variants (i.e. internal allele frequency < 0.1% and an external allele frequency in gnomAD < 0.003%). *S*_het_ burden scores for PTVs have a skewed distribution and most individuals in the general population have scores close to zero, whereas only a small percentage have scores exceeding 0.6 ( Figure 6).

In each birth cohort, we selected a variable that captures educational attainment, school performance, or cognitive ability, and examined the association between these and *S*_het_ burden in a subset of children predicted to have European ancestry (using projection in 1000 Genomes space) who passed exome QC. For ALSPAC, we chose the variable YPF7980, which measures the years that the individual spent in education around 26 years of age (ALSPAC child-completed ‘Life@26+’ questionnaire, see YPF file), and performed the analysis in 2,631 probands. In MCS, we tested the association between *S*_het_ burden and ‘word reading test’ scores measured at age 7 (variable DCWRSC00) in 5,354 children. In BiB, we used the English reading test scaled scores from Key Stage 2 (KS2) UK national curriculum tests in 790 children. We acknowledge that these samples and variables are not necessarily representative of the diversity of the cohorts and the myriad of possible traits measured in these cohorts; however, they were chosen because we had a reasonable expectation of a positive association with PTVs and no association with synonymous variants. The findings were interpreted only in a technical context to see whether the QC was adequate.

The association between the dependent variables and *S*_het_ burden was tested in a regression model, using sex and three principal components (projections in 1000 Genomes space) as covariates. The regression analysis was performed using the glm() function from the stats package in R 4.1.0. The variable YPF7980 (ALSPAC) is a discrete numeric variable (years in education). To simplify the modelling, we dichotomised this variable to indicate whether the Young Person in ALSPAC had more than 13 education-years (coded as 1/0, so that 1 represents having >13 years of education, which is roughly equivalent to having a higher education) and used logistic regression to test the association with *S*_het_ burden. The word reading test scores from MCS and the English reading test scores from BiB were rank-normalised using the RankNorm() function from RNOmni package in R, and their association with *S*_het_ burden was tested using ordinary least-square linear regression.

Using these models, the regression coefficient in ALSPAC reflects the odds of having more than 13 years of education that is associated with an *S*_het_ burden score of 1. In MCS and BiB, it reflects the change in the reading test score (in standard deviations) that is seen with an *S*_het_ burden score of 1. In practice, no one has an *S*_het_ burden of 1 for high-confidence, damaging PTVs, with most people have scores closer to 0, whereas most individuals have an *S*_het_ burden close to 1 for ultra-rare synonymous variants ( Figure 6) ( Gardner *et al.*, 2022).

***Assessing the role of DNA source in ALSPAC***

Background: *The DNA used for sequencing in a substantial percentage of ALSPAC parents came from immortalised cell lines, whereas the DNA from the children was derived from peripheral white blood cells (from blood samples). Cell lines are known to accumulate mutations as they are grown and maintained. Often, these mutations are not seen in all cells in a cell line (i.e. they are “mosaic”) and may resemble sequencing artefacts. We were concerned that the DNA derived from cell lines might harbour somatic mutations that had occurred and been subject to positive selection in cell culture. These would appear as rare variants, and would be more likely to be protein-truncating or missense variants as they have functional effects. To ensure that our QC is sufficient to harmonise the quality of variants identified across samples with different DNA sources, we compared the number of variants in individuals whose source of DNA was blood to the number of variants in those whose sample was from a cell-line.*

The individuals were stratified by DNA source and the counts were compared between the two groups. In total, we studied 675 unrelated individuals with cell-line derived DNA (parents) and 8,709 individuals with blood-derived DNA (children). For each gene, the carriers of rare synonymous variants and rare PTVs variants (allele frequency 0.1% in ALSPAC and gnomAD) were counted after applying the three different variant/genotype filters (relaxed, medium, stringent), each coupled with three missingness filters (no filtering on missingness, removing variants with ≥ 50% samples with missing calls after applying the filters, or removing those with ≥ 5% missingness). Fisher's exact test was performed to test the significance of the difference in the number of carriers between blood- and cell line-derived DNA for each gene-filter combination. We applied an exome-wide significance cut-off accounting for 18 tests (2 consequence classes x 3 QC stringencies x 3 missingness filters) per each of ~ 20,000 protein-coding genes (p<1.38x10 ^-7^).

## Results

### Evaluating the effect of QC stringency on exome-wide rare variant burden associations

Background: *Rare deleterious protein-truncating variants (PTVs) in so-called “loss-of-function-intolerant” genes are under negative selection in the general population and have known effects on several human traits, particularly those related to cognitive function. To evaluate the effect of different QC stringencies on rare variant association testing, we calculated scores representing the total burden of extremely rare, deleterious PTVs amongst participants with ES data in the birth cohorts after applying different QC thresholds, and tested the association of these burden scores with selected phenotypes related to school performance and educational attainment.*

The distribution of *S*_het_ burden scores for ultra-rare PTVs and synonymous variants is shown in Figure 6. In ALSPAC, higher *S*_het_ burden of PTVs was significantly associated with lower educational attainment, specifically, lower odds of having >13 education-years, whereas in MCS and BiB, higher *S*_het_ burden of PTVs was significantly associated with lower reading test scores ( Figure 6a). Filtering stringency had only a very small impact on the effect size and significance of these PTV associations in ALSPAC and BiB, with more stringent filtering generally resulting in a slightly attenuated effect size in ALSPAC and a slightly larger effect size in BiB, whereas in MCS, all estimates of effect size were similar. Missingness filters did not have a noticeable effect. Generally, the differences between the results obtained with the different QC thresholds were extremely subtle and not significant. There was no significant association with synonymous *S*_het_ burden in any cohort (a negative control; Figure 6b).

### The role of DNA source in ALSPAC

Background: *To assess any difference in mutational burden between cell-line-derived and blood-derived samples in ALSPAC, and how that might vary with the different QC stringencies, we compared the counts of individuals with and without a rare variant in each autosomal protein coding gene, assessing synonymous and high confidence PTVs filtered with different levels of stringency.*

Reassuringly, the *p*-values from our gene-based rare variant burden tests did not differ substantially from those expected from a uniform distribution (Extended Data Figure 7a). The top-ranked gene in both synonymous and protein-truncating variants analysis was *IGLL5*, which codes for the immunoglobulin lambda-like polypeptide 5 (immunoglobulin light chain). The *p*-values for the relaxed and medium filters ( *p*_Synonymous_ = 8.6x10 ^-6^; *p*_Protein-truncating_ = 1.18x10 ^-4^ ) were more significant than those observed with the stringent filters for this gene ( *p*_Synonymous_ = 0.029; *p*_Protein-truncating_ = 0.0013) but not significant after correction for multiple testing (p > 1.38x10 ^-7^), neither did they exceed the expected 95% confidence interval. Filtering on the genotyping rate did not have a substantial effect on the observed *p-*values.

Thus, the QC filters seem adequate to remove somatic mutations that were subject to positive selection in cell lines in single-gene comparisons. However, this does not rule out that the samples derived from cell lines in ALSPAC may have sporadic, randomly-distributed somatic mutations that likely appear as rare variants. To explore this further, we regressed the variant counts per sample on the DNA source and three principal components to estimate how many additional PTVs cell line-derived samples harbour on average, relative to blood-derived samples. We found that the stringent set of filters resulted in PTV counts that were not significantly different between the two groups (p=0.0502, with the 5% missingness filter; Extended Data Figure 7b). Synonymous variants were more frequent in cell-line derived samples, even when using stringent QC (p<1.2x10 ^-4^), with these harbouring one to two more variants on average than blood-derived samples (Extended Data Figure 7b). This suggests that cell-line derived samples do have a slight excess of rare variants which probably result from somatic mutations, so users may wish to control for DNA source when conducting analyses, to be conservative.

***Using birth cohorts to estimate the genetic prevalence of disease***

*Background: In addition to association analyses with binary and quantitative traits, birth cohorts offer the chance to examine the genetic prevalence of monogenic disorders (i.e. the prevalence of pathogenic variants in the relevant causal gene fitting with the relevant mode of inheritance). The genetic prevalence approaches the population prevalence of an inherited disease if all or most disease-causing variants can be accurately identified. As a positive control, we tested whether we see the expected number of children harbouring homozygous disease-causing variants in CFTR, the causative gene for Cystic Fibrosis.*

Cystic Fibrosis is a severe recessive single-gene disease caused by loss-of-function variants in *CFTR*. It is generally more prevalent amongst those of European genetic ancestry ( Bobadilla *et al*., 2002; Ideozu *et al*., 2024; Southern *et al*., 2007). The symptoms typically start before 2 years of age (e.g. pronounced respiratory difficulties and infections) and the median age at death in the UK is ~38 years ( The Cystic Fibrosis Trust, 2022). We evaluated the genetic prevalence of Cystic Fibrosis in 17,031 maximally unrelated children of European genetic ancestry (ALSPAC: 7,766; MCS: 6,049; BiB: 3,216). For simplicity, we evaluated the frequency of variants reported as pathogenic or likely pathogenic in ClinVar and did not consider possible compound heterozygous alleles since this could only be done using the subset of children in trios. 

In the UK, Cystic Fibrosis is usually diagnosed through newborn screening and has an incidence of 1 in 2,500 live births ( The Cystic Fibrosis Trust, 2022). We expect 7 children with homozygous pathogenic *CFTR* variants amongst 17,031 live births from all three cohorts (the sample size divided by 2,500, rounded to the nearest integer). The 95% confidence interval for the genetic prevalence is 3 – 14 diagnoses (Poisson distribution). We found 3 children with known homozygous pathogenic variants ( *p*-value = 0.1803, Poisson test). These children harboured Phe508del, the commonest pathogenic allele in the UK ( Ideozu *et al*., 2024), and the genotypes passed all QC stringencies.

## Conclusions and recommendations

Here, we describe the production, quality control and validation of exome sequencing datasets encompassing more than 37,000 individuals from three birth cohorts (ALSPAC = 11,651; BiB = 11,659; MCS = 14,592). The variant calling focused on SNVs and short indels. We have performed several analyses to validate the overall quality of the final datasets. We therefore anticipate that most users will not need to repeat the QC on these datasets if they only want to study SNVs and small indels, but can work with the post-QC version we released (see Data Availability). For standard analyses, we recommend using the stringent QC and filtering for variants with a minimum genotyping rate of 50% ( Table 3). However, experienced users may, of course, prefer to take the raw data (see Data Availability) and employ different QC, depending on the balance of sensitivity/specificity they wish to achieve for a given analysis or whether, for example, they wish to combine a cohort with another dataset. To assist with such endeavours, the released VCFs contain the reference GIAB sample GIAB12878/HG001 we used to estimate precision and recall, in addition to four other GIAB samples (Extended Data Table 4). We note that it may be possible to produce a better balance of sensitivity and specificity for the ALSPAC dataset by applying different QC thresholds to C>A/G>T SNVs versus other SNVs, but have not explored this ourselves yet.

These data will be most valuable to decipher the relationship between rare variants and various phenotypes (see example in Figure 6), especially traits measured at multiple time points ( Malawsky *et al*., 2024) and diseases that have onset in adolescence or (as the cohorts age) early adulthood. Analyses of severe neonatal or childhood disorders that may bias participation should be attempted with care (e.g., by verifying that the rate of reported diagnoses and genetic prevalence in the birth cohort are consistent with known disease rates). Notably, we found that the genetic prevalence of Cystic Fibrosis when examined collectively across all three cohorts (amongst maximally unrelated children of European ancestry) is not substantively different from the reported disease prevalence in the UK. Despite this, users should of course exercise caution when studying disease rates for rare conditions in a single cohort (e.g., to ensure that they are sufficiently prevalent to be detected with confidence). Another important factor to consider when examining genetic prevalence of inherited diseases is the age at which the samples used for DNA extraction were collected, since participants with severe developmental disorders that present before these ages may have been less likely to give DNA.

In particular, the current collection might not be well-suited to examine severe, rare and early-onset conditions linked to newly arising mutations. The QC we have employed is likely not optimal for *de novo* mutations, so a separate release of those employing a different, tailored QC process will be made available at a later date. Similarly, copy-number variants will be called based on these exome sequence data, and subject to appropriate QC, then the calls released. Users who are interested in studying additional difficult-to-call variant types (e.g. short tandem repeats ( Halman & Oshlack, 2020), or medium-sized indels ( Gardner *et al.*, 2021)) will need to carry out their own bespoke variant calling and QC in order to optimally capture those variants. Users should also consider accounting for DNA source in their analysis of the ALSPAC data (e.g. using it as a covariate in a regression model or as a stratum in exact tests) or performing additional QC and harmonisation.

While for this initial data release, variant calling and QC of these three cohorts has been conducted separately, we plan to carry out joint calling and QC of these three cohorts (plus additional samples which are being sequenced) together over the next year, in order to facilitate harmonisation of the datasets. Until that dataset is available, we caution against attempting to aggregate individual-level data across cohorts in mega-analyses, or comparing the numbers of variants in particular classes between datasets. Rather, meta-analysing the results of analyses carried out separately on the different cohorts is likely to be a safer approach.

We expect these new ES datasets of British birth cohorts to become a useful resource for the global research community. We are continuing to produce additional ES data from these and other cohorts, and this data note will be adapted as new data become available.

## Data Availability

The genetic data from this study have been deposited in the European Genome-Phenome Archive (EGA). For each birth cohort, the released data includes raw exome sequence data (sample-level CRAM files including those failing our QC) plus a multi-sample project VCF (pVCF) of all samples passing all stages of sample QC, split by chromosome. See ‘Accessing Restricted Data’ and ‘Usage Notes’ below for the details and Figure 1 for the sample sizes. We have also released sample-level genetic measures, namely, predicted genetic ancestry labels shown in Figure 2, principal component eigenvectors (projected in 1000 Genomes space), exome-wide variant counts of ultra-rare damaging PTVs (see Dataset validation for the definition) and synonymous variants, as well as *S*_het_ burden scores based off of these variants. These are available through applications to the Data Access Committees of the respective cohorts, as detailed below. ***Accessing restricted data*** The EGA study accession numbers are: ALSPAC (study: EGAS00001005273): dataset EGAD00001015371 MCS (study: EGAS00001007789): dataset EGAD00001015372 BiB (study: EGAS00001006978): dataset EGAD00001015370 Eligible applicants can access the exome sequencing data through applications to the EGA: https://ega-archive.org/access/request-data/how-to-request-data/ Further guidance on the process to access EGA is explained on the website of the European Bioinformatics Institute: https://www.ebi.ac.uk/training/online/courses/ega-quick-tour/accessing-the-data-in-the-ega/ Requests to access the linked data (e.g., phenotypes, clinical and educational records, genetic burden scores, other omics) should be directed to the individual cohorts as detailed below (see *Access regulation*). ***Usage notes*** •   The data release includes pVCFs (VCFv4.2) produced using Hail’s export_vcf(); These are provided as bgzip compressed chromosome-level VCF files aligned to GRCh38. •   Each released pVCF includes the cohort samples that passed all sample QC steps ( Figure 1) along with 5 reference samples from the GIAB consortium (Extended Data Table 4). •   While we recommend applying the stringent filters, we included all the variants that pass the relaxed QC filters to allow for more flexibility, and annotated the percentage of sample passing the relaxed, medium, and stringent QC filters as INFO fields. Similarly, the genotypes were not filtered but we rather indicated whether each genotype passes each of the three filters in the FORMAT field. These annotations related to the QC are detailed in Extended Data Table 5. Multi-allelic variants were split. •   Additionally, the variant consequences on Ensembl genes and gnomAD allele frequencies were annotated using VEP (Extended Data Table 6). •   We have provided guidance on applying the recommended filters as well as a few examples for common use scenarios in the supplementary, e.g. counting variants, locating specific variants, and calculating burden scores (see Extended Data). ***ALSPAC*** The ALSPAC study website contains details of all the data that is available through a fully searchable data dictionary and variable search tool: http://www.bristol.ac.uk/alspac/researchers/our-data/ ALSPAC omics data (e.g., ES, genotype data) is described in: https://www.bristol.ac.uk/alspac/researchers/our-data/genomics-data/ Variant calls from these exome data will be available as a standard omics dataset by ALSPAC, with raw files available through EGA (dataset EGAD00001015371). All samples which were found to be different from the array sample were excluded from the standard dataset, but the raw exome files will be available as a non-standard data request from ALSPAC. Full details of the ALSPAC consent procedures are available on the study website: http://www.bristol.ac.uk/alspac/researchers/research-ethics/ Data access is possible through a system of managed open access. The steps below highlight how to apply for access to the data included in this data note and all other ALSPAC data: i. Please read the ALSPAC access policy, which describes the process of accessing the data and samples in detail, and outlines the costs associated with doing so: http://www.bristol.ac.uk/media-library/sites/alspac/documents/researchers/data-access/ALSPAC_Access_Policy.pdf ii. You may also find it useful to browse our fully searchable research proposals database, which lists all research projects that have been approved since April 2011: https://proposals.epi.bristol.ac.uk/?q=proposalSummaries iii. Please submit your research proposal for consideration by the ALSPAC Executive Committee. https://proposals.epi.bristol.ac.uk/ iv. You will receive a response within 10 working days to advise you whether your proposal has been approved. ***MCS*** The MCS is conducted by and housed at the Centre for Longitudinal Studies (CLS) at University College London. The MCS website, with documentation for the cohort and detailed information about current research and publications, is available at: https://cls.ucl.ac.uk/cls-studies/millennium-cohort-study/ Information on genetic and other omic data is available at: https://cls-genetics.github.io/ Variant calls from this exome data will be available as a standard omics dataset via EGA (dataset EGAD00001015372). The MCS data are available without charge to bona fide researchers under standard access conditions. To link exome data with other phenotype / survey data collected in MCS, researchers should apply via CLS DAC which meets monthly: https://cls.ucl.ac.uk/data-access-training/data-access/accessing-data-directly-from-cls ***BiB*** The BiB website contains further details about the study, links to detailed data dictionaries, and information about how to apply for access to data: https://borninbradford.nhs.uk/ Variant calls from this exome data will be available as a standard omics dataset via EGA with the dataset accession number EGAD00001015370. Researchers are encouraged to make applications to use BiB data. Applications can be made via an expression of interest form available on the study website: https://borninbradford.nhs.uk/research/how-to-access-data/ Figshare: Supplementary to the Data Note: Exome Sequencing of UK Birth Cohorts, https://doi.org/10.6084/m9.figshare.25907992.v1 ( Koko *et al.*, 2024). This project contains the following extended data: Extended_Glossary.pdf (glossary of technical terms used in the Data Note (related to ES and QC) and their explanations) Extended_Supplementary.pdf (notes about the Birth Cohorts, Extended Data Figures 1–7, Extended Data Tables 1–6) Extended_Usage_Examples.html (examples for common use cases and pointers on using the released pVCFs for basic queries) Twist_NGSTECustom_001418_target_padded_100bp.bed.zip (GRCh38 genomic coordinates of Twist custom exome enrichment targets (padded, used for variant calling)) Data are available under the terms of the Creative Commons Attribution 4.0 International license (CC-BY 4.0). NEBNext® Ultra™ II DNA Library Prep Kit: https://international.neb.com/products/e7645-nebnext-ultra-ii-dna-library-prep-kit-for-illumina#Product%20Information Twist Standard Hybridization Reagent Kit v2: https://www.twistbioscience.com/products/ngs/reagent-kits/Standard-Hybridization-Reagent-Kit

## Software availability

The software used for variant calling and quality control are available from these repositories:

- GATK: https://github.com/broadinstitute/gatk/releases
- Hail: https://hail.is/
- VEP: https://hub.docker.com/r/ensemblorg/ensembl-vep
- bcftools: https://samtools.github.io/bcftools/bcftools.html
- PLINK: https://www.cog-genomics.org/plink/
- R: https://cran.r-project.org/

Additional documentation for the QC methods implemented in Hail and documentation for various aspects of exome quality control from the genome aggregation database (gnomAD), which was largely followed in this analysis, is available here:

- https://hail.is/docs/0.2/methods/genetics.html#hail.methods.sample_qc
- https://github.com/wtsi-hgi/wes-qc/
- https://broadinstitute.github.io/gnomad_methods/
- https://gnomad.broadinstitute.org/news/2018-10-gnomad-v2-1/
- https://gnomad.broadinstitute.org/news/2019-10-gnomad-v3-0/
- https://gnomad.broadinstitute.org/news/2020-10-gnomad-v3-1-new-content-methods-annotations-and-data-availability/
