# Supplementary material for: Exome sequencing of UK birth cohorts
Source: Wellcome Open Res. 2024 Dec 5;9:390. Originally published 2024 Jul 19. [Version 2] doi: 10.12688/wellcomeopenres.22697.2 (PMC11747307; doi:10.12688/wellcomeopenres.22697.2)
Supplement: Peer review history

# Reviewer report: Reviewer response for version 2

**DOI:** 10.21956/wellcomeopenres.25892.r114821

**Published:** 2025-01-30

**Authors:** Kristiina Tammimies

**Affiliations:** 1 Karolinska University Hospital, Solna, Sweden; 2 Karolinska Institute Department of Women's and Children's Health, Stockholm, Stockholm County, Sweden

This data note by Koko et al. presents quality-controlled whole exome sequencing (WES) data derived from three UK birth cohorts: the Avon Longitudinal Study of Parents and Children (ALSPAC), the Millennium Cohort Study (MCS), and Born in Bradford (BiB). These cohorts have been earlier genotyped and have provided information on common variant contributions to health, development, and life-course trajectories, which now can be expanded to WES data. The ALSPAC cohort includes over 8,400 children and 3,200 parents, predominantly of white ethnicity, while the MCS includes more than 7,600 children and 6,900 parents from a nationally representative sample enriched for ethnic diversity. The BiB cohort consists of over 8,700 children and nearly 2,900 parents, with a significant proportion of Pakistani participants, reflecting Bradford's population's diversity.

 The work presents a comprehensive framework for quality control of WES data, effectively addressing sequencing artifacts and minimizing batch effects across these cohorts. The data analysis and processing pipeline are highly robust, and the descriptions and sharing of results are more than adequate.

 The authors demonstrate how the data can be utilized by analyzing the impact of rare and ultra-rare variants on educational and cognitive traits. Additionally, they estimate the prevalence of the rare genetic condition Cystic Fibrosis, as requested by a reviewer.

 This data note offers a detailed description of the WES data from three UK birth cohorts. However, the following areas could be clarified or expanded to improve its utility for researchers:

  

1. As noted by the other reviewer, the data note would benefit from some brief demographic information for the participants with WES data, such as sex/gender and socioeconomic factors. This could be added to Table 1.
2. It would be highly useful to note the number of fully sequenced trios (mother, father, child) and mother/father-child dyads within each cohort. This information is critical for researchers planning to investigate de novo mutations, genetic nurture effects, and other parent-offspring genetic interactions. This information could also be added to Table 1.

Are sufficient details of methods and materials provided to allow replication by others?

Yes

Is the rationale for creating the dataset(s) clearly described?

Yes

Are the datasets clearly presented in a useable and accessible format?

Yes

Are the protocols appropriate and is the work technically sound?

Yes

Reviewer Expertise:

neurodevelopmental disorders, genetics

I confirm that I have read this submission and believe that I have an appropriate level of expertise to confirm that it is of an acceptable scientific standard.

# Reviewer report: Reviewer response for version 2

**DOI:** 10.21956/wellcomeopenres.25892.r114823

**Published:** 2025-01-20

**Authors:** Ahmad Aziz

**Affiliations:** 1 DZNE - German Center for Neurodegenerative Diseases, Bonn, Germany

Koko *et al*.  provide quality-controlled WES data from three UK birth cohorts: the Avon Longitudinal Study of Parents and Children (8,436 children and 3,215 parents), the Millenium Cohort Study (7,667 children and 6,925 parents) and Born in Bradford (8,784 children and 2,875 parents). They provide a detailed and comprehensive overview of the methodology used for sequencing as well as for the extensive QC pipeline. Overall, I think that the authors have done an excellent job; access to these WES data of these three UK birth cohorts will be very useful to the global research community.

**I have only some minor comments:**

- Although the authors provide brief descriptions of each cohort and provide links to the primary sources, it would be helpful to provide a tabular/graphical overview of these cohorts, especially including more demographic/phenotypic data on each cohort (e.g., distributions of age, sex, educational attainment in each cohort).
- The QC has been performed for each cohort separately. It would be helpful to provide an integrated QC across the three cohorts, although I do understand that this is not trivial and could also be added at a later stage after the release of the cohort specific WES data.
- It is reassuring that the genetic prevalence of CF based on these UK birth cohorts parallels known prevalence estimates in the general UK population. The authors found 3 CF cases; it would be helpful to indicate to which of these birth cohorts these 3 cases belonged to.

Are sufficient details of methods and materials provided to allow replication by others?

Yes

Is the rationale for creating the dataset(s) clearly described?

Yes

Are the datasets clearly presented in a useable and accessible format?

Yes

Are the protocols appropriate and is the work technically sound?

Yes

Reviewer Expertise:

epidemiology, genetics, multiomics, neurology, neuroscience

I confirm that I have read this submission and believe that I have an appropriate level of expertise to confirm that it is of an acceptable scientific standard.

# Reviewer report: Reviewer response for version 1

**DOI:** 10.21956/wellcomeopenres.24998.r101307

**Published:** 2024-10-23

**Authors:** Jan M Friedman

**Affiliations:** 1 University of British Columbia, British Columbia, Canada

Koko and associates describe the production, rigorous quality control, and validation of exome sequencing datasets from three UK birth cohorts that together include nearly 25,000 children and more than 13,000 of their parents. The project identified almost 2,000,000 SNVs and more than 52,500 short indels that appear to be true positives, as well as about 3 times as many SNVs and about 6 times as many indels that are probably false positives. All exome sequencing data from this study are being publicly released as sample-level CRAM files, along with a combined file of all variants from samples that passed all stages of quality control and an extensive set of metadata. Future data releases will include copy number variants and high-confidence *de novo* variants.

Are sufficient details of methods and materials provided to allow replication by others?

Yes

Is the rationale for creating the dataset(s) clearly described?

Yes

Are the datasets clearly presented in a useable and accessible format?

Yes

Are the protocols appropriate and is the work technically sound?

Yes

Reviewer Expertise:

Clinical genomics

I confirm that I have read this submission and believe that I have an appropriate level of expertise to confirm that it is of an acceptable scientific standard.

# Reviewer report: Reviewer response for version 1

**DOI:** 10.21956/wellcomeopenres.24998.r101308

**Published:** 2024-10-23

**Authors:** Stephen Kingsmore

**Affiliations:** 1 Rady Children's Institute for Genomic Medicine, San Diego, California, USA

This Data Note describes whole exome sequencing data from three UK birth cohorts: the Avon Longitudinal Study of Parents and Children (8,436 children and 3,215 parents), the Millenium Cohort Study (7,667 children and 6,925 parents) and Born in Bradford (8,784 children and 2,875 parents). The objective of this effort is to make the resulting high-quality data widely accessible to the global research community.

 93% of ALSPAC vcfs passed QC, as did 97% of BiB vcfs, and 95% of MCS. All of these are excellent.

 Of concern was that of 9 million variants in ALSPAC, 5 million (54%) were FPs, compared with 10% in MCS and 17% in BiB. The ALSPAC variant FP rate is very high, and the BiB number is concerning. As the authors note in the discussion, these QC methods are not optimal for de novo or ultra-rare variants. Given this concern it would be important to measure the genetic prevalence of one or two rare, severe childhood genetic diseases in the cohorts to determine whether it is consistent with the birth prevalence of those disorders. Cystic fibrosis would be a good example. This would reassure readers of the quality of the data for severe, childhood genetic disease research. Without such reassurance it is likely that the ALSPAC dataset should be used only with substantial caution. Of note, the paper does not mention the age at which samples were obtained in the 3 cohorts which is critical for understanding the expected prevalence of rare, severe childhood genetic diseases.

Are sufficient details of methods and materials provided to allow replication by others?

Yes

Is the rationale for creating the dataset(s) clearly described?

Yes

Are the datasets clearly presented in a useable and accessible format?

Yes

Are the protocols appropriate and is the work technically sound?

Yes

Reviewer Expertise:

Genomics, genetics, childhood genetic diseases, molecular diagnosis

I confirm that I have read this submission and believe that I have an appropriate level of expertise to confirm that it is of an acceptable scientific standard, however I have significant reservations, as outlined above.

# Response

**Published:** 2024-11-22

**Authors:** Mahmoud Koko

**Affiliations:** Human Genetics, Wellcome Sanger Institute, Hinxton, England, UK

> This Data Note describes whole exome sequencing data from three UK birth cohorts: the Avon Longitudinal Study of Parents and Children (8,436 children and 3,215 parents), the Millenium Cohort Study (7,667 children and 6,925 parents) and Born in Bradford (8,784 children and 2,875 parents). The objective of this effort is to make the resulting high-quality data widely accessible to the global research community. 93% of ALSPAC vcfs passed QC, as did 97% of BiB vcfs, and 95% of MCS. All of these are excellent.  

**Response: **We are very thankful for the positive reception of this work and the feedback on aspects needing clarification. We have updated the data note to address the presented comments as we detail below. We also corrected a few typos throughout the text and added a link under ‘Software availability’ for a public Git repository containing the code used for the QC.  

 > Of concern was that of 9 million variants in ALSPAC, 5 million (54%) were FPs, compared with 10% in MCS and 17% in BiB. The ALSPAC variant FP rate is very high and the BiB number is concerning.  

**Response: **We acknowledge that the FP rate in ALSPAC before QC is very high due to a sequencing artefact. However, we emphasise that the goal of the QC was to remove these false positives while retaining true positives, and we believe we showed that it achieved this goal fairly effectively. Generally, we think FP rates after QC are more relevant than before QC, since in practice, users will be working with post-QC data.   As we discuss in Box 3, the excess number of SNVs in ALSPAC results from a sequencing artefact (C>A/G>T) that has certain contexts and patterns (discussed in Costello et al., 2013 PMID: 23303777). This results in FPs having much lower quality metrics (e.g., FisherStrand score), explaining why we were able to eliminate the vast majority of them through QC. We found that binned random forest score cut-offs ~40 in ALSPAC will retain most TPs while cleaning up most FPs (shown in Figure 2). To demonstrate this, we provided an overview of TP/FP rates for three representative random forest thresholds in each cohort (relaxed, medium, stringent) in Extended Data Figure S4. This figure shows that the FP rate will drop significantly to < 3% with any reasonable QC, with the TP rate still maintained above 90% (including in ALSPAC).   We show in Table 3 the performance metrics after applying the set of stringent QC filters we recommend for standard use. The FP rate after this QC was 0.1% in ALSPAC (~5,000/5 million FPs retained) vs. 0.3% in MCS (~1,300/~430,000 FPs retained) and 0.5% in BiB (~1,000/~200,000 FPs retained). We also noted in the discussion that it may be possible to produce even a better balance of sensitivity and specificity for the ALSPAC dataset by applying more stringent QC thresholds to C>A/G>T SNVs versus other SNVs, although we have not attempted this ourselves.   Regarding BiB, both the TP and FP counts (pre-QC) in BiB were (mistakenly) given in the text as 674,752; This is indeed the correct TP count before QC whereas the correct FP count is 198,711 (FP rate < 5% before QC rather than 17%). This has been amended in the text.    Given that the QC seemed to reduce the FPs to what we believe are acceptable levels, we decided that relying on this variant QC was more sensible than resequencing all sample batches showing the C>A artefact (the complete ALSPAC cohort and part of MCS), which would have cost around $1 million.

 > As the authors note in the discussion, these QC methods are not optimal for de novo or ultra-rare variants.   

**Response: ** While we acknowledge that the QC is not optimal for de novo mutations (as it was not meant to be, since they require special treatment), we did not say that the QC methods are not optimal for ultra-rare variants and we suspect there may be a misunderstanding here. We have shown in Fig. 5 (now Fig. 6) that ultra-rare variants are adequately QCed, showing the expected associations with a few selected education-related traits. Therefore, we believe that prospective users can utilise these data for various analyses given that they appropriately consider other sources of variability in ultra-rare variant counts (e.g. genetic ancestry).    For more clarity, we added a new figure (now Fig. 5) depicting summary statistics of rare (frequency < 0.1% in gnomAD) and ultra-rare (< 0.003% in gnomAD) variant counts in the three cohorts after applying the recommended filters. In this plot we show that the median counts and interquartile ranges are comparable amongst the three birth cohorts between individuals with predicted European genetic ancestry and also individuals with other ancestries. This shows that, after QC and once restricting to European-ancestry samples, the three cohorts look very similar in terms of the distribution of ultra-rare synonymous and protein-truncating variants per person. This is reassuring that the QC was adequate in removing the vast number of FPs in ALSPAC, and that the three cohorts look similar after QC.

 See Figure 5 linked here https://wellcomeopenresearch.s3.eu-west-1.amazonaws.com/linked/360866.Mahmoud_Koko.pdf 

 Figure 5: Rare and ultra-rare variant counts in the birth cohorts. The boxplots show the median and interquartile range (IQR) after applying the recommended filters shown in Table 3. Two (gnomAD) allele frequency thresholds are shown (rare: 0.1%; ultra-rare 0.003%). The whiskers show the range excluding (solid lines) or including outliers (dashed lines). The largest sample groups in terms of predicted genetic ancestry (European (EUR) in the three cohorts and South Asian (SAS) in BiB) are plotted separately from the remaining samples.

 > Given this concern it would be important to measure the genetic prevalence of one or two rare, severe childhood genetic diseases in the cohorts to determine whether it is consistent with the birth prevalence of those disorders. Cystic fibrosis would be a good example. This would reassure readers of the quality of the data for severe, childhood genetic disease research. Without such reassurance it is likely that the ALSPAC dataset should be used only with substantial caution. Of note, the paper does not mention the age at which samples were obtained in the 3 cohorts which is critical for understanding the expected prevalence of rare, severe childhood genetic diseases.

**Response: ** Thank you for the suggestion. We have now added the age information to the methods and extended the results and discussion to address the genetic prevalence of Cystic Fibrosis as a form of positive control. Given that the incidence is variable across ancestry groups, this analysis was limited to a set of maximally unrelated children of predicted European genetic ancestry across all cohorts. However, we did not present a cohort-level analysis to comply with the approved QC proposal - particularly in relation to the use of identifiable data (in a single cohort, counts less than 5 are considered identifiable).

 Specifically, a paragraph explaining the age at which samples were obtained was added in the Methods:   The age at which biological samples were collected varied across cohorts. Whereas BiB collected umbilical cord blood at birth (< 500 children were sampled later), for this project, around one third of ALSPAC child samples were taken from those collected at birth and two thirds from around the age of 7 years; Saliva sampling for MCS was performed around age 14.   We added a new subsection at the end of the ‘Data validation’ section   Using birth cohorts to estimate the genetic prevalence of disease   Background: In addition to association analyses with binary and quantitative traits, birth cohorts offer the chance to examine the genetic prevalence of monogenic disorders (i.e. the prevalence of pathogenic variants in the relevant causal gene fitting with the relevant mode of inheritance). The genetic prevalence approaches the population prevalence of an inherited disease if all or most disease-causing variants can be accurately identified. As a positive control, we tested whether we see the expected number of children harbouring homozygous disease-causing variants in CFTR, the causative gene for Cystic Fibrosis. 

 Cystic Fibrosis is a severe recessive single-gene disease caused by loss-of-function variants in CFTR. It is generally more prevalent amongst those of European genetic ancestry (Bobadilla et al. 2002; Southern et al. 2007; Ideozu et al. 2024). The symptoms typically start before 2 years of age (e.g. pronounced respiratory difficulties and infections) and the median age at death in the UK is ~38 years (The Cystic Fibrosis Trust, 2022). We evaluated the genetic prevalence of Cystic Fibrosis in 17,031 maximally unrelated children of European genetic ancestry (ALSPAC: 7,766; MCS: 6,049; BiB: 3,216). For simplicity, we evaluated the frequency of variants reported as pathogenic or likely pathogenic in ClinVar and did not consider possible compound heterozygous alleles since this could only be done using the subset of children in trios.    In the UK, Cystic Fibrosis is usually diagnosed through newborn screening and has an incidence of 1 in 2,500 live births (The Cystic Fibrosis Trust, 2022). We expect 7 children with homozygous pathogenic CFTR variants amongst 17,031 live births from all three cohorts (the sample size divided by 2,500, rounded to the nearest integer). The 95% confidence interval for the genetic prevalence is 3 - 14 diagnoses (Poisson distribution). We found 3 children with known homozygous pathogenic variants (p-value = 0.1803, poisson test). These children harboured p.Phe508del, the commonest pathogenic allele in the UK (Ideozu et al. 2024), and the genotypes passed all QC stringencies.   The following was added to the ‘Conclusions and Recommendations’ section (now 2nd and beginning of 3rd paragraph):   These data will be most valuable to decipher the relationship between rare variants and various phenotypes (see example in Figure 6), especially traits measured at multiple time points (Malawsky et al., 2024) and diseases that have onset in adolescence or (as the cohorts age) early adulthood. Analyses of severe neonatal or childhood disorders that may bias participation should be attempted with care (e.g., by verifying that the rate of reported diagnoses and genetic prevalence in the birth cohort are consistent with known disease rates). Notably, we found that the genetic prevalence of Cystic Fibrosis when examined collectively across all three cohorts (amongst maximally unrelated children of European ancestry) is not substantively different from the reported disease prevalence in the UK. Despite this, users should of course exercise caution when studying disease rates for rare conditions in a single cohort (e.g., to ensure that they are sufficiently prevalent to be detected with confidence). Another important factor to consider when examining genetic prevalence of inherited diseases is the age at which the samples used for DNA extraction were collected, since participants with severe developmental disorders that present before these ages may have been less likely to give DNA.    In particular, the current collection might not be well-suited to examine severe, rare and early-onset conditions linked to newly arising mutations; [Paragraph continues: The QC we have employed is likely not optimal for de novo mutations...]

 References:   Bobadilla JL, Macek M Jr, Fine JP, Farrell PM. Cystic fibrosis: a worldwide analysis of CFTR mutations--correlation with incidence data and application to screening.

 Hum Mutat. 2002;19(6):575-606. doi:10.1002/humu.10041  

 Ideozu JE, Liu M, Riley-Gillis BM, et al. Diversity of CFTR variants across ancestries characterized using 454,727 UK biobank whole exome sequences. Genome Med. 2024;16(1):43. Published 2024 Mar 21. doi:10.1186/s13073-024-01316-5  

 Malawsky DS, Koko M, Danacek P, et al: The differential effects of common and rare genetic variants on cognitive performance across development. medRxiv. 2024. doi: 10.1101/2024.09.04.24313061  

 The Cystic Fibrosis Trust. The Addendum For the 2011 Standards for the Clinical Care of Children and Adults with Cystic Fibrosis in the UK guideline. 2022. Reference source: https://www.cysticfibrosis.org.uk/sites/default/files/2022-10/Standards%20of%20care_interim%202022.pdf   

 Southern KW, Munck A, Pollitt R, et al. A survey of newborn screening for cystic fibrosis in Europe. J Cyst Fibros. 2007;6(1):57-65. PMID: 16870510. doi:10.1016/j.jcf.2006.05.008
